# Concurrent OPA1 mutation and chromosome 3q deletion leading to Behr syndrome: a case report

**DOI:** 10.1186/s12887-020-02309-0

**Published:** 2020-09-03

**Authors:** Ting Zeng, Linyan Liao, Yi Guo, Xuxu Liu, Xiaobo Xiong, Yu Zhang, Shi Cen, Honghui Li, Shuzhang Wei

**Affiliations:** 1 Department of Child Healthcare, Liuzhou Maternity and Child Healthcare Hospital, 50 Boyuan Avenue, Liuzhou, 545616 China; 2 Key Laboratory of Developmental Disorders in Children, Liuzhou Maternity and Child Healthcare Hospital, 50 Boyuan Avenue, Liuzhou, 545616 China

**Keywords:** OPA1, Optic atrophy, Behr syndrome, Microdeletion, Case report

## Abstract

**Background:**

Optic atrophy 1 (OPA1) gene mutations are associated with dominantly inherited optic neuropathy resulting in a progressive loss of visual acuity. Compound heterozygous or homozygous variants that lead to severe phenotypes, including Behr syndrome, have been reported rarely.

**Case presentation:**

Here, we present a 14-month-old boy with early onset optic atrophy, congenital cataracts, neuromuscular disorders, mental retardation, and developmental delay. Combined genetic testing, including whole exome sequencing (WES) and chromosomal microarray analysis, revealed a concurrent *OPA1* variant (c.2189 T > C p.Leu730Ser) and de novo chromosome 3q deletion as pathogenic variants leading to the severe phenotype.

**Conclusions:**

Our case is the first reporting a novel missense *OPA1* variant co-occurring with a chromosomal microdeletion leading to a severe phenotype reminiscent of Behr syndrome. This expands the mutation spectrum of *OPA1* and inheritance patterns of this disease.

## Background

The optic atrophy 1 (OPA1) gene encodes a dynamin-related protein located within the mitochondrial inner membrane, and is involved in multiple critical cellular and mitochondrial functions [1]. Variants in the *OPA1* gene are mostly related with autosomal dominant optic atrophy (DOA) (OMIM#165500) [2–5]. This disorder is characterized by an insidious onset of visual impairment in early childhood with moderate to severe loss of visual acuity, temporal optic disc pallor, color vision deficits, and centrocecalscotoma of variable density. Dominant optic atrophy plus (DOA+) syndrome is observed in 20% of patients with pathogenic *OPA1* variants [4–6], manifesting extra-neuromuscular features like ataxia, myopathy, peripheral neuropathy, sensorineural deafness, and chronic progressive external ophthalmoplegia.

To date, over 500 pathogenic variants have been documented in *OPA1* genes that are inherited in a dominant manner, with an estimated prevalence at 1/50,000 [7]. *OPA1* variants with a recessive inheritance mode that cause the Behr syndrome have been recently revealed in a few cases with early-onset optic atrophy [8, 9], spinocerebellar degeneration, mental retardation, and developmental delay [10]. Clinical heterogeneity has been observed in patients with compound heterozygous or homozygous variants of the *OPA1* gene [11]. Here, we report a boy with a hemizygous *OPA1* variant unmasked by concurrent chromosome 3q deletion. The patient showed early onset and progressive optic atrophy, peripheral neuropathy, and developmental delay resembling Behr syndrome. This case could help extend the phenotypic and genotypic spectrum of the disorder.

## Case presentation

### Clinical data

A 14-month-old boy was referred to the department of Developmental Behavior Pediatrics at Liuzhou Maternity and Child Healthcare Hospital, Guangxi province, for developmental delay, Achilles tendon contracture, hypertonia, nystagmus, and vision defects. The boy was born at full term following an uneventful pregnancy to non-consanguineous parents of Chinese descent. Neither of the parents nor their related family members had any related symptoms.

**Fig. 1:**
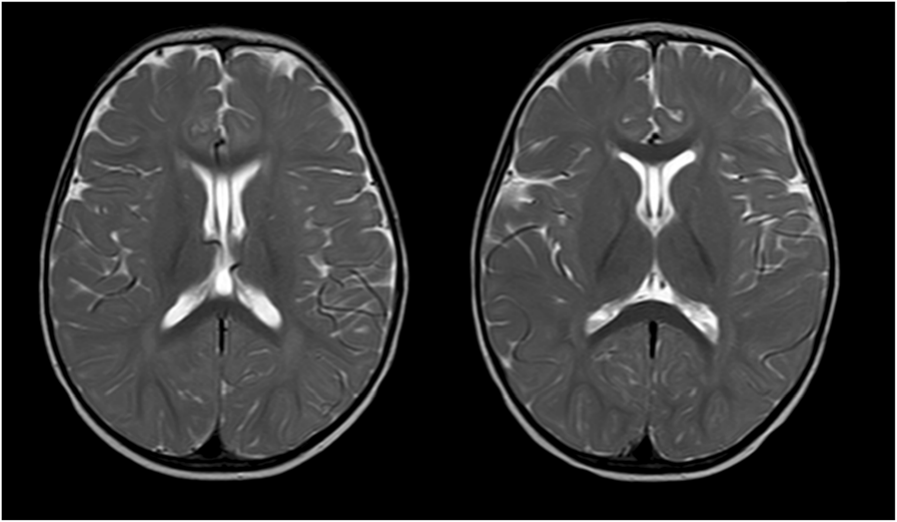
Brain magnetic resonance images at 14 months of age. The images show delayed myelination for this age

The boy had normal birth length and weight (52 cm and 3.3 kg) and is now 80 cm and 10.8 kg (update to 17/4/2019). The parents described the boy failing to track light or objects, and nystagmus was noticed since birth. Ophthalmological examination revealed no eye tracking, horizontal tremor of both eyes, exotropia, and corneal transparency. Congenital cataracts were found in the patient. No abnormalities were found in the ophthalmological evaluations for the father of the proband. Developmental milestones were obviously delayed. He could not roll over until 12 months, or get to a sitting position without assistance until 18 months. The boy has had hypertonia and Achilles tendon contracture since birth. The Gesell developmental test was performed and the boy was evaluated a shaving severe developmental delay of adaptability and fine motor skills, moderate developmental delay of large motor skills, and mild developmental delay of language development and personal-social interactions. Magnetic resonance imaging revealed delayed myelination of the brain and formation of the fifth ventricle (Fig. 1). Karyotyping showed normal 46, XY. Tandem mass spectrometry was applied to detect possible inherited metabolic diseases for the proband but with negative results.Genetic testing and data analysis.

Genomic DNA was extracted from peripheral blood samples of the patient and his parents using the GentraPuregene Blood Kit (Qiagen, Hilden, Germany) according to the manufacturer’s protocol. Proband-only targeted next-generation sequencing using an inherited disease panel (including 2742 disease-causing genes, cat No. 5190–7519, Agilent Technologies, Santa Clara, CA, USA) was performed as described previously [12]. Original sequencing data were assessed using Fast QC (version 0.11.2) for quality control. The Burrows-Wheeler Alignment tool (version 0.2.10) was employed for aligning sequencing data to the Human Reference Genome (NCBI build 37, hg 19). Single nucleotide variants and small indels were identified by the Genome Analysis Toolkit. All variants were saved in Variant Call Format and uploaded to the Ingenuity Variant Analysis (Ingenuity Systems, Redwood City, CA, USA) and TGex (Translational Genomics Expert) platforms for biological analysis and interpretation. Variants detected by next-generation sequencing were confirmed by Sanger sequencing in the patient and his parents.

Copy number variants (CNVs) were identified using CNVkit open source software, a tool kit to infer and visualize copy numbers from targeted DNA sequencing data. Aligned data after the Burrows-Wheeler Alignment process were used as input. Normal reference data used for CNV identification were constructed using sequencing data from 10 normal males and 10 normal females that were previously validated without pathogenic CNVs by a chromosomal microarray platform. Default CNVkit settings were used for CNV identification. CNVs detected through bioinformatics analysis are further validated by chromosomal array analysis using CytoScan 750 k Suite (Thermo Scientific, Waltham, MA, USA).

**Fig. 2:**
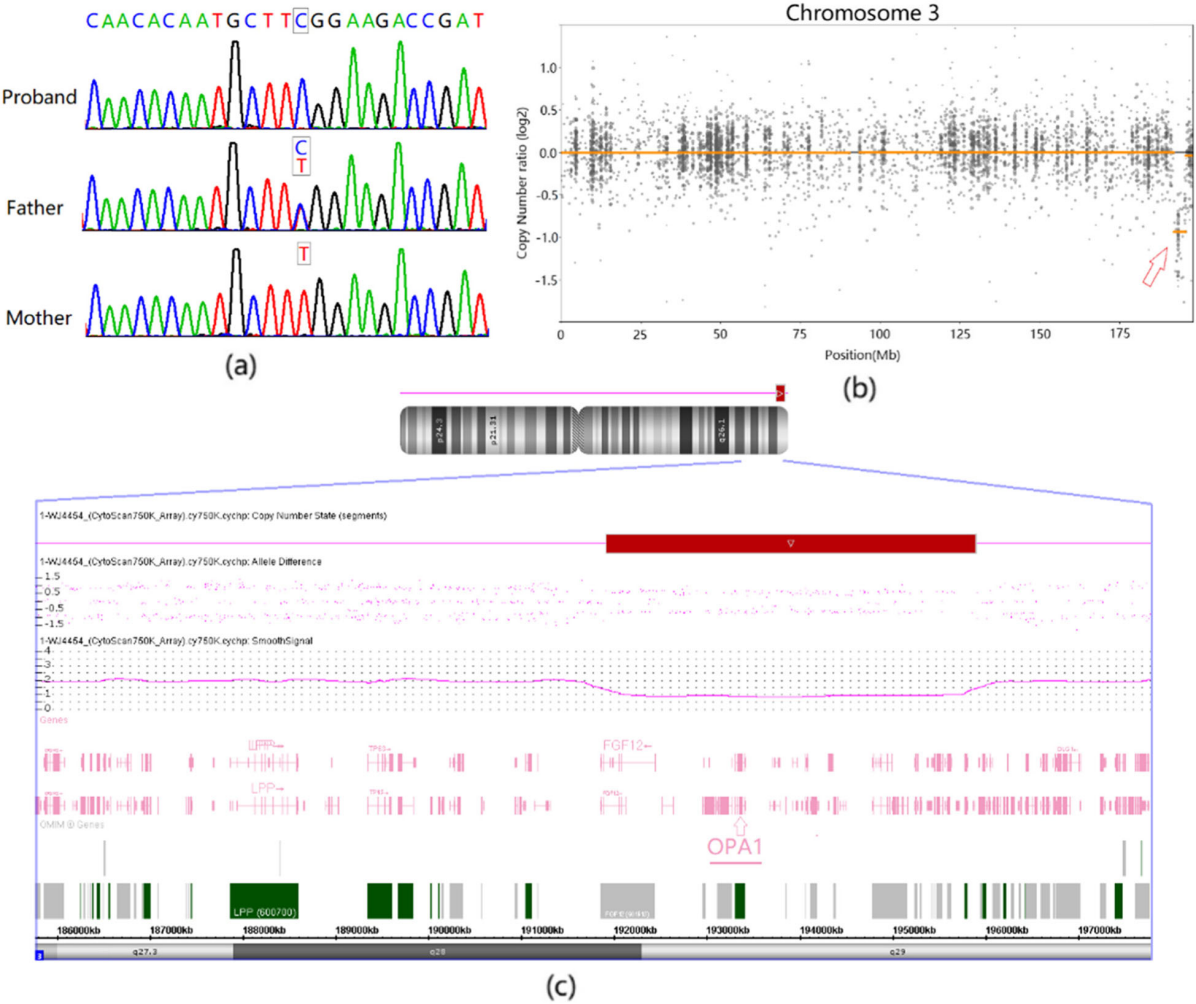
**a** Sanger sequencing of the proband and his parents showed that the proband was homozygous, the father heterozygous, and the mother wild-type. **b** Sketch map of the chromosome 3 copy number state for the proband from the CNVkit. The microdeletion is indicated by a red arrow with the copy number ratio at − 1.0. **c** Chromosomal microarray analysis precisely mapping the deletion on 3q28q29 and covering the OPA1 gene

A homozygous variant in *OPA1* (NM_015560: c.2189 T > C p.Leu730Ser) was identified in the patient. The variant is absent from all currently available databases including the Genome Aggregation Database. Multiple lines of computational evidence suggest a deleterious effect of this mutation (Table 1). Sanger sequencing was applied to validate the variant in the pedigree and revealed that the patient’s father carried the same heterozygous variant and his mother was wild-type (Fig. 2a). CNV analysis, based on read-depth information of the proband, indicated a heterozygous deletion on the short arm of chromosome 3 (Fig. 2b). Chromosomal microarray analysis confirmed this deletion as arr[GRCh37] 3q28q29(191921285_195896838)× 1(Fig. 2c). Thus, the proband inherited a heterozygous variant in *OPA1* gene from his father and developed a de novo 3975 kb microdeletion on chromosome 3 that unmasked the heterozygous variant into a hemizygous form.

**Table 1:** In-silico damaging effect prediction of missense variants

Softwares/Algorithms	Score	Deleterious/Pathogenicity threshold
Clin Predict	0.99	> 0.5
REVEL	0.92	> 0.5
M-CAP	0.39	> 0.11
ReVe	0.99	> 0.66
VEST3	0.97	> 0.61
FATHMM	−4.00	≤ − 0.92
SIFT	0.00	≤0.01
PolyPhen2-HDIV	1.00	> 0.93
PolyPhen2-HVAR	0.99	> 0.62
GERP++	5.89	> 3.86
CADD	6.33	> 23.95

## Discussion and conclusion

Heterozygous mutation of the *OPA1* gene, which encodes a multifunctional protein located within the mitochondrial inner membrane leading to optic atrophy under a dominant inheritance pattern, has been well-defined and identified in hundreds of patients. Ban et al. [13] reported that *OPA1* disease alleles associated with DOA displayed selective defects in several activities, including cardiolipin association, GTP hydrolysis, and membrane tabulation. Twenty percent of those patients with only one *OPA1* mutation manifested a DOA+ phenotype, presenting extra-ocular manifestations. Yu-Wai-Man et al. [5] revealed that there was a 2- to 4-fold increase in the mitochondrial DNA copy number at the single-fiber level, and greater mitochondrial DNA proliferation in cytochrome coxidase-negative skeletal muscle fibers induced by *OPA1* mutations in patients with DOA+ features.

Compound heterozygous mutations in *OPA1* have been reported in a few patients with DOA+, or even more severe and early onset phenotypes. Schaaf et al. reported two siblings presenting severe DOA+ features and identified biallelic *OPA1* mutations in these individuals [14]. Each parent was heterozygous for one of the mutations and also manifested mild related phenotypes. This indicated clinical heterogeneity of those mutations and a possible dosage effect of the OPA variants. Alessia et al. [15] reviewed reported cases with biallelic *OPA1* variants and found that most patients carried the p.Ile437Met missense variant that was considered hypomorphic, or with very low potential pathogenicity. The p.Ile437Met missense variant did not result in clinical symptoms of autosomal DOA in the heterozygous or homozygous states. Thus, the p.Ile437Met missense variant was thought to contribute consistently to modulating the phenotype in OPA1 compound heterozygous subjects, suggestive of either recessive or semi-dominant patterns of inheritance in those DOA+ patients with biallelic OPA1 variants.

The Behr syndrome is characterized by early-onset optic atrophy, ataxia, pyramidal signs, peripheral neuropathy, mental retardation, and developmental delay [8, 16]. Bonneau et al. [8] reported four unrelated children with a phenotype consistent with Behr syndrome. Three of the four patients carried a truncating mutation on one allele and the same missense mutation (Ile382Met) on the other allele. Co-occurrence of a truncating *OPA1* mutation with another missense mutation may result in a dominant-negative effect and a more severe phenotype, particularly if the missense mutation occurs in the GTPase domain of the protein. Only two patients with homozygous missense mutations in OPA1 have been reported to date [17, 18]. The missense mutation identified in our patient was first recognized as homozygous, but later proved to be hemizygous by further definition of the concurrent chromosomal microdeletion. This is thought to be extremely rare compared with reported dominant or recessive inheritance modes, thus rapid molecular analysis revealing underlying pathogenic variants is of great importance in those confusing clinical case.

Genomic rearrangements, including larger deletions or duplications, are frequent in patients with optic atrophy [19]. This indicates that the hypothesis that haploinsufficiency could be a major pathologic mechanism in *OPA1* mutations associated with autosomal DOA [20]. Although the 3975 kb deletion also covered disease-related genes *FGF12* and *TFRC*, none of them was an established haploinsufficiency gene or with reported pathogenic deletions in patients. To our knowledge, all patients with exonic or chromosomal deletions encompassing *OPA1* exhibited symptoms consistent with OPA without any neurologic involvement. Thus, the severe neuromuscular and developmental phenotypes in our patient further supported the recessive pathogenic mode in our proband. Optic atrophy was evident in all reported patients with DOA, DOA+, and Behr syndrome, being often the first and main symptom. Our patient showed early onset optic atrophy, congenital cataracts, neuromuscular disorders, mental retardation, and developmental delay reminiscent of Behr syndrome, expanding the mutation spectrum of *OPA1*. The genetic findings in this unique case also emphasized the importance of CNVs analysis in diagnosis protocol of Behr syndrome.

In conclusion, we report a unique case of optic atrophy with a severe neuromuscular phenotype and a recessive inheritance mode. The missense variant, c.2189 T > C p.Leu730Ser, detected in the proband has not been reported previously in DOA-related phenotypes. A concurrent chromosomal deletion in the proband contributed to the pathogenesis of a phenotype reminiscent of Behr syndrome in a recessive mode with rare missense variants. This rare case will help in further exploring and understanding the detailed pathologic mechanism in OPA1 patients.

## Data Availability

Data used during the current study are available from the corresponding author upon reasonable request.

## Abbreviations

OPA: Optic atrophy
WES: Whole exome sequencing
DOA: Dominant optic atrophy
CNVs: Copy number variants
GTP: Guanosine triphosphate
